# Clinical and medication profiles stratified by household income in patients referred for diabetes care

**DOI:** 10.1186/1475-2840-6-11

**Published:** 2007-03-30

**Authors:** Doreen M Rabi, Alun L Edwards, Lawrence W Svenson, Peter M Sargious, Peter Norton, Erik T Larsen, William A Ghali

**Affiliations:** 1 Department of Medicine, University of Calgary, Calgary, Canada; 2 Department of Community Health Sciences, University of Calgary, Calgary, Canada; 3 Centre for Health and Policy Studies, University of Calgary, Calgary, Canada; 4 Department of Family Medicine, University of Calgary, Calgary, Canada; 5 Alberta Health and Wellness, Edmonton, Canada; 6 Calgary Laboratory Services, Calgary, Canada

## Abstract

**Background:**

Low income individuals with diabetes are at particularly high risk for poor health outcomes. While specialized diabetes care may help reduce this risk, it is not currently known whether there are significant clinical differences across income groups at the time of referral. The objective of this study is to determine if the clinical profiles and medication use of patients referred for diabetes care differ across income quintiles.

**Methods:**

This cross-sectional study was conducted using a Canadian, urban, Diabetes Education Centre (DEC) database. Clinical information on the 4687 patients referred to the DEC from May 2000 – January 2002 was examined. These data were merged with 2001 Canadian census data on income. Potential differences in continuous clinical parameters across income quintiles were examined using regression models. Differences in medication use were examined using Chi square analyses.

**Results:**

Multivariate regression analysis indicated that income was negatively associated with BMI (p < 0.0005) and age (p = 0.023) at time of referral. The highest income quintiles were found to have lower serum triglycerides (p = 0.011) and higher HDL-c (p = 0.008) at time of referral. No significant differences were found in HBA1C, LDL-c or duration of diabetes. The Chi square analysis of medication use revealed that despite no significant differences in HBA1C, the lowest income quintiles used more metformin (p = 0.001) and sulfonylureas (p < 0.0005) than the wealthy. Use of other therapies were similar across income groups, including lipid lowering medications. High income patients were more likely to be treated with diet alone (p < 0.0005).

**Conclusion:**

Our findings demonstrate that low income patients present to diabetes clinic older, heavier and with a more atherogenic lipid profile than do high income patients. Overall medication use was higher among the lower income group suggesting that differences in clinical profiles are not the result of under-treatment, thus invoking lifestyle factors as potential contributors to these findings.

## Background

Individuals with low income are at increased risk for the development of diabetes [1-3] Low income is also an independent predictor of hospitalization for the acute complications of diabetes and is associated with higher odds of microvasculopathy and heart disease [4-6].

There is an extensive literature that explores the association between income and health outcomes among the general population. The relationship between income and health outcomes is complex and is mediated by a number of factors. Potential mediating factors include differential access to care [7-10], behavioural and psycho-social factors [11-16], and biologic factors [17-19]. Several researchers have shown, that even within universal health care systems, the poor are less effective at accessing specialty care [7-10,20]. They are also more likely to partake in poor health-related behaviours (such as smoking, consuming diets low in fruits and vegetables, and sedentary lifestyles [20-23]) and are more likely to be overweight or obese [22,24,25]. Biologic differences among income groups have also been identified. High income groups are more likely to have higher levels of HDL cholesterol and low income groups have been found to have subtle changes in their neuro-endocrine and immune responses that may predispose them to atherosclerosis [17-19,26].

Low income patients with diabetes are at greater risk for adverse health outcomes but the factors influencing this relationship are unclear. There is emerging evidence that income does not appear to effect access to specialty diabetes care [27,28], but little is known about clinical, behavioural or biologic differences across income groups, among those with diabetes.

In recognizing our incomplete understanding of the income relationship to diabetes, this study proposed to explore whether there are clinical and/or biologic differences across income groupings among patients referred to an urban diabetes education centre (DEC). The study's objectives specifically included an assessment of the clinical profiles (including medication use) of patients across income groupings at the time of referral for specialized diabetes care.

## Methods

### Data Sources

To conduct this work, we used a regional DEC database that captures basic demographic information on all attendees to the regional clinic situated in Calgary, Alberta, a large Canadian city. The sampling frame was all active patients at the DEC from May 1, 2000 to January 9, 2002. The sample consisted of 4687 patients. All patients included were from a single health region within the province of Alberta. This DEC is the single regional provider of diabetes education services. Access is dependent upon physician referral to the centre. The postal codes of patients registered in the DEC database were linked to their corresponding dissemination area (DA) using the Statistics Canada Postal Code Conversion File (PCCF).

Neighborhood income data were obtained from Statistics Canada Census data (2001). We defined a neighborhood as equivalent to a census dissemination area (DA)- a small geographic area covered by a single census data collector which typically contains 400–700 persons. Therefore, median household income per DA was the income measure used in this study. These data were merged with the DEC database on the variable DA. Neighbourhood income has been shown to be reasonably concordant with individual income in urban settings [29,30]. There is also increasing evidence that neighbourhood income is valid SES construct that predicts health outcomes independently of individual income [31,32].

### Derivation of Income Quintiles

Household income quintiles were generated from DA annual income data. All income data is reported in Canadian dollars. The size and associated incomes for the income quintiles were as follows:

1) Income quintile 1, n = 940, less than $40877

2) Income quintile 2, n = 937, $40878 – $53065

3) Income quintile 3, n = 936, $53066 – $62921

4) Income quintile 4, n = 938, $62922 – 79828

5) Income quintile 5, (n = 936), more than $79829

### Study Variables and Statistical Analyses

Physicians referring patients to the DEC complete a standardized referral form that includes clinical data. This information was then entered into the DEC patient registry. Clinical information examined in this study included: serum hemoglobin A1C (HBA1C); serum lipid profiles including levels of low density lipoprotein (LDL-c), high density lipoprotein (HDL-c) and triglyceride; microalbumin to creatinine ratios and medications used at time of referral. Height and weight are measured upon presentation to clinic; these measures were used to calculate the body mass index (BMI) which was then entered into the DEC database.

Potential differences in continuous clinical parameters across income quintiles were examined using regression models. If inspection of the distribution of these variables suggested a linear relationship between income and the variable of interest, then income quintile was modeled as a single ordinally-coded predictor variable. If, on the other hand, the relationship was not linear, then regression was performed using dummy variables for each income quintile relative to the lowest income quintile as a reference group. Covariates considered in these models included sex and medication use. Differences in categorically-coded medication use across income quintiles, meanwhile, were examined using Chi square analyses. All statistical analyses were performed in STATA, version 8.

## Results

### Descriptive Analysis

**Figure 1:**
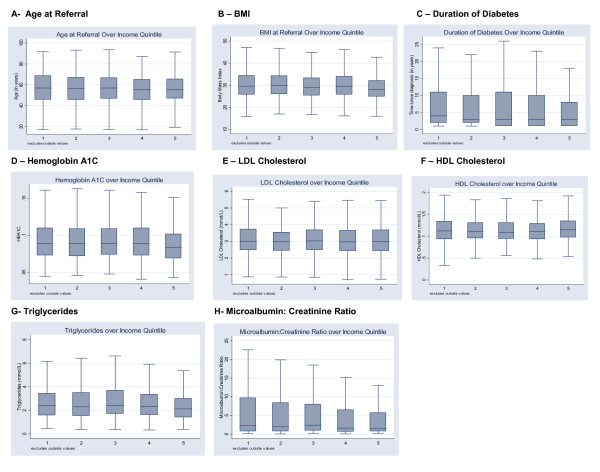
Distribution of clinical variables across income quintiles.

Clinical characteristics of patients referred for diabetes care and education are listed, by income quintile, in Table 1. The median age of patients increased as income level decreased. The median age in the highest income groups (quintile 5) was 55.3 years compared to the lowest income group (quintile 1) which was almost 2 years older at time of referral with a median age of 57.0 years. There was a similar inverse relationship between income level and BMI. The median BMI in the wealthiest quintile (quintile 5) was 28 compared median BMIs of 29.6 in quintile 1 and 29.8 in quintile 2. Our results also indicate that the lowest income quintile presents to clinic later from the time of diagnosis of diabetes. The median duration of diabetes was 4 years in the lowest quintile, compared to 3 years in all of the other quintiles. In terms of diabetes-related clinical parameters, patients did not differ significantly across groups with respect to serum LDL-c levels. Patients in the highest income quintile had the highest HDL-c. An inverse relationship between income and triglycerides is suggested as the median triglyceride levels range from 2.40 mmol/L in the lowest income group down to 2.12 mmol/L in the highest income group. Glycemic control appears to be slightly better in the highest income group as evidenced by a median HBA1C of 8.4% in quintile 5 compared to a median HBA1C of 8.9% in all other income groups. There is also a suggestion of a negative association between microalbumin creatinine ratio (M:C) and income. The lowest income group had a median M:C of 2.3 compared to a median M:C of 1.5 in the highest income group. Boxplots illustrating the distribution of clinical characteristics are illustrated in figure 1.

**Table 1:** Clinical Profiles at time of referral by income quintile

	**Income Quintile**					
	
	**1 (low)**	**2**	**3**	**4**	**5 (high)**	*p*-for trend*
**Clinical Characteristic**	Median (IQR)					

Age (in years)	56.95 (22.9)	56.52 (21.48)	56.95 (19.94)	55.24 (19.44)	55.27 (18.5)	0.032
BMI	29.6 (8.6)	29.8 (8.3)	29 (7.9)	29.5 (8.2)	28 (7.2)	<0.0005
Duration of Diabetes (in years)	4 (9)	3 (8)	3 (10)	3 (9)	3 (7)	0.98
LDL-c (mmol/L)	3.01 (1.26)	2.97 (1.1)	3.02 (1.22)	2.97 (1.23)	2.99 (1.26)	**
HDL-c (mmol/L)	1.12 (0.41)	1.1 (0.36)	1.09 (0.37)	1.1 (0.36)	1.15 (0.35)	**
Triglycerides (mmol/L)	2.40 (1.88)	2.29 (1.99)	2.41 (2.0)	2.32 (1.76)	2.12 (1.61)	**
HBA1C (%)	8.9 (3.7)	8.9 (3.7)	8.9 (3.5)	8.9 (3.7)	8.4 (3.3)	**
Microalbumin: Creatinine	2.3 (9)	1.95 (7.7)	2.4 (7.1)	1.6 (5.9)	1.5 (5)	**

*results of univariate analyses for trend when income could be modeled as a single, ordinally-coded variable

**these variables did not have a simple linear relationship with income (results of regression models where income quintiles are modeled as dummy variables are displayed in Table 3).

### Regression Analysis

#### Clinical profiles

Visual inspection of the distribution of the variables age, body mass index (BMI), and duration of diabetes (Figure 1, panels a, b and c), suggested a linear relationship between those clinical characteristics and income. Linear regression on ordinally coded income groupings was thus performed (see Table 2) and indeed demonstrated a significant negative association between age at the time of referral and median household income per DA (β-coefficient = -.339, 95% CI -0.65 – -0.03). A similar negative association was noted between BMI and income where the β-coefficient was found to be -.362 (95% CI -0.51 – -0.21). This relationship remained significant even after controlling for age and diabetes medication use. These findings reveal that wealthy patients presenting for diabetes education are younger and leaner than those from lower income groups. While this might suggest that the wealthy are presenting earlier in the course of their diabetes, we found no significant association between income and the duration of diabetes (β-coefficient=-.0167, 95% CI -1.32 – 1.29).

**Table 2:** Association of Income Quintile with General Clinical Parameters

**Clinical Characteristic**	**Co-variate**	**B-coefficient (p-value)**	**B-coefficient – adjusted for sex (p-value)**	**B-coefficient – adjusted for sex, age & therapy (p-value)**
Age	Quintile	-.339 (0.032)	-.361 (0.023)	
BMI	Quintile	-.362 (<0.0005)	-.319 (<0.0005)	-.306 (<0.0005)
Duration of DM	Quintile	-.0167 (0.980)	.0272 (0.968)	

Visual inspection of the distribution on the clinical variables of LDL-c, HDL-c, triglycerides, HBA1C and microalbumin:creatinine ratio did not reveal an obvious linear relationship in the associations with income (Figure 1, panels d-h). In this instance, regression modeling was done by comparing each individual income quintile to a pre-defined reference group, income quintile 1. Table 3 lists the results of the analysis. No significant association was found between income and level of glycemic control as measured by HBA1C. After controlling for age, sex, and differences in the use of anti-diabetic medications, the highest income quintile had a trend towards a lower HBA1C. Similarly, while no significant association was found with respect to microalbumin:creatinine ratio, the box plot of this variable (Figure 1, panel h) suggests that the highest income groups have lower ratios.

**Table 3:** Association of Income Quintile with Diabetes-related Clinical Parameters

**Clinical Parameter**	**Income Quintile**	**B-coefficient – Unadjusted (p-value)**	B-coefficient – Adjusted (p-value)*
HDL-C	1 (reference)		
	2	.0126 (0.798)	.015 (0.76)
	3	.0625 (0.201)	.063 (0.20)
	4	.0151 (0.757)	.021 (0.67)
	5	.0756 (0.120)	.084 (0.09)
LDL-c	1 (reference)		
	2	-.0813 (0.222)	-.086 (0.193)
	3	-.010 (0.885)	-.016 (0.805)
	4	-.0391 (0.540)	-.044 (0.488)
	5	-.022 (0.731)	-.025 (0.691)
HDL-c	1 (reference)		
	2	-.013 (0.52)	-.01 (0.6)
	3	-.018 (0.35)	-.009 (0.64)
	4	-.014 (0.46)	-.001 (0.95)
	5	.031 (0.11)	.05 (0.008)
Triglycerides	1 (reference)		
	2	-.23 (0.40)	-.22 (0.43)
	3	-.05 (0.86)	-.06 (0.84)
	4	-.26 (0.34)	-.28 (0.31)
	5	-.63 (0.019)	-.68 (0.011)
Microalbumin: Creatinine	1 (reference)		
	2	-8.56 0.058	-8.97 (0.047)
	3	-2.88 0.540	-2.43 (0.602)
	4	-7.58 0.097	-7.16 (0.116)
	5	-6.99 0.122	-7.01 (0.119)

*Adjusted for age, sex, and therapy (HBA1C was adjusted for anti-hyperglycemic medication use, LDL-c, HDL-c and Triglycerides were adjusted for lipid-lowering therapy use and Microalbumin: creatinine was adjusted for anti-hypertensive medication use.)

The association of serum lipid levels at the time of referral was also examined. While no relationship was found between the levels of LDL-c and income quintile, significant findings were noted with respect to HDL-c and triglyceride levels. In the unadjusted analysis, HDL-c was highest in the wealthiest income quintile but this did not reach statistical significance. After adjusting for differences in sex, age and use of lipid lowering medications, the association strengthened and became significant. Triglyceride levels were similarly lowest in the highest income group, and this was significant both in the unadjusted and adjusted analyses (Table 3).

#### Medication Use

The proportions of patients, by income quintile, prescribed specific medications are presented in Table 4. Chi square analyses indicate socio-economic gradients for the use of certain diabetes therapies. A statistically significant gradient was noted for the use of diet alone to manage diabetes. In the lowest income quintile, 14.4% of patients presented on diet alone, compared to 24.4% in the highest income group (χ^2 ^= 44.22, *p *< 0.0005). An inverse gradient was noted in the use of oral diabetes medications. Metformin was used by 37.3% of patients in the lowest income group, compared to 30% in the highest income group (χ^2 ^= 18.85, p = 0.001). Sulfonylureas were also more commonly used in the lower income quintiles compared to the highest income quintiles (χ^2 ^= 25.63, p < 0.0005). No significant differences were found across income quintiles in the use of glucosidase inhibitors (χ^2 ^= 2.99, p = 0.558), thiazolideindiones (TZD) (χ^2 ^= 2.93, p = 0.087) or subcutaneous insulin (χ^2 ^= 2.56, p = 0.392).

**Table 4:** Association of Income with Medical Therapy Use

	**Income Quintile**					***P*-value**
		
	**1 (low)**	**2**	**3**	**4**	**5 (high)**	
**Therapy**						

Diet Only	14.1%	14.4%	16.9%	18.8%	24.4%	<0.0005
Metformin	37.3%	36.1%	37.0%	31.5%	30.0%	0.001
Sulfonylureas	29.6%	30.1%	29.3%	24.1%	22.4%	<0.0005
Glucosidase Inhibitors	2.0%	1.3%	1.8%	2.0%	1.2%	0.558
TZD	3.9%	3.5%	4.1%	4.2%	3.5%	0.886
Insulin	19.8%	18.2%	18.3%	18.6%	17.2%	0.688
Lipid Lowering Medication	11.8%	9.0%	9.3%	9.6%	11.0%	0.212
Anti-Hypertensive Medication	19.7%	21.7%	19.0%	19.7%	19.0%	0.596

## Discussion

Individuals with low income and diabetes are at increased risk for developing vascular complications. While the processes mediating this low income/poor health outcome relationship have been examined in the general population, little is known about the factors mediating this relationship among those with diabetes. Previous research has shown that access to specialty diabetes care appears equitable across income groups [27,28], suggesting that differences in health outcomes may be mediated by other factors.

### Clinical and Biologic Factors

This study demonstrates that there are clinically significant differences in some biologic parameters across income quintiles and that low income patients present to clinic with higher risk profiles. Low-income patients are older at time of referral and have more atherogenic metabolic profiles with higher serum triglycerides and lower HDL levels, which are associated with a higher risk for developing cardiovascular disease [33-35]. While a significant association between income and HBA1C was not noted, inspection of the distribution of HBA1C suggests a trend towards a lower HBA1C in the highest income quintile.

This study also suggests that differences in metabolic status are not due to overt under-treatment of the economically disadvantaged. The lowest income groups were using more sulfonylureas and metformin compared to the wealthiest groups. Even the use of more costly therapies such as TZD and lipid-lowering therapies were similar across income groups.

### Behavioural Factors

This study also provides some insight into potential health related behavioural differences across income groups. It has been shown in previous research that sedentary lifestyles are more common among lower income populations. In this study, the lower income groups had the highest BMIs. This raises the possibility that the lower income groups are less physically active than their wealthy counterparts. The lower HDL levels and higher triglycerides might also reflect behavioural differences with respect to diet and/or exercise [36]. Unfortunately, the data set used in this study does not contain detailed data on diet or exercise so we were unable to explicitly assess differences in these important behaviours.

### Other considerations

High income is frequently associated with higher health literacy and a greater ability to apply health-related knowledge [37,38]. It should be noted that while we did not find that HBA1Cs differ significantly at the time of referral, others have documented that individuals from higher socio-economic strata are more likely to experience significant lowering of their HBA1Cs after assessment at diabetes clinic [37,38]. It would be most interesting to know had the clinical profiles of these patients been re-evaluated 1 year following their referral whether the differences noted would have remained the same, been attenuated or perhaps been even more pronounced due to differences in health literacy.

While we did not find a significant difference with respect to the duration of the diagnosis of diabetes at the time of referral, examination of the distribution of this variable certainly suggests that this may, in part, be mediating some of clinical differences noted. The wealthiest patient group was also younger, and more likely to be controlled with diet alone, suggesting that these patients may be presenting at an earlier point in the natural history of their diabetes. If wealthy patients were being referred earlier (perhaps due to earlier diagnosis), this may also help explain the inverse relationship between income and complication risk. As there is now clear evidence that aggressive management of blood glucose, high blood pressure and high serum lipids will effectively prevent the micro- and macrovascular complications of diabetes [39-43], it follows that the earlier a specialist intervenes, the more effective these prevention strategies might be.

This study has limitations. This is a cross sectional study that examined the clinical profiles of patients at one point in time. These referrals were not necessarily index referral, and had we compared clinical profiles at first contact with specialty care, it is possible that some of the clinical differences noted may have been attenuated. It is noteworthy that clinical data were entered into the DEC database from a standardized clinic referral form. All clinical data examined in this study, therefore, were provided by the referring physician. If doctors differ in the manner in which they complete, or do not complete this form, an information bias could be introduced to this study. We do not have any evidence, however, that physicians' documentation skills should differ based on the neighbourhood income of their patients, and would assert that information bias relating to income is unlikely.

## Conclusion

This study provides important information on how the clinical profiles of patients with diabetes differ based on income. Given that elevated serum lipids, HBA1C and microalbumin to creatinine ratios are all significant predictors of atherosclerosis and mortality [43-45], it is quite plausible that these clinical differences mediate the relationship between income and health outcomes in this population. Whether these differences are influenced by patient, physician, or other factors, remains unclear. However, this study does provide reassurance that within Canada's single payer health care system, prescribing practices do not appear to discriminate against individuals of lower income. In fact, overall mediation use was higher in the lower income groups, appropriately reflecting their higher burden of vascular risk factors.

## Competing interests

All listed authors would like to declare that there were no competing interests involved with this research or the preparation of this manuscript.

## Authors' contributions

DMR conceived the study. DMR and WAG collaborated on the study design. WAG, ALE, PMS, PN and ETL were all involved in the establishment of the database used in this study. DMR led the writing of this manuscript but all listed authors contributed substantially to the editorial process and approved the final manuscript.
